# Psychotropic in the environment: risperidone residues affect the behavior of fish larvae

**DOI:** 10.1038/s41598-017-14575-7

**Published:** 2017-10-26

**Authors:** Fabiana Kalichak, Renan Idalencio, João Gabriel Santos da Rosa, Heloísa Helena de Alcântara Barcellos, Michele Fagundes, Angelo Piato, Leonardo José Gil Barcellos

**Affiliations:** 1 Programa de Pós-Graduação em Farmacologia, Universidade Federal de Santa Maria (UFSM), Av. Roraima, 1000, Cidade Universitária, Camobi, Santa Maria, RS 97105-900 Brazil; 2 Universidade de Passo Fundo (UPF), BR 285, São José, Passo Fundo, RS 99052-900 Brazil; 3 Programa de Pós-Graduação em Ciências Ambientais, Instituto de Ciências Biológicas, Universidade de Passo Fundo (UPF), BR 285, São José, Passo Fundo, RS 99052-900 Brazil; 4 Programa de Pós-Graduação em Farmacologia e Terapêutica, Instituto de Ciências Básicas da Saúde, Universidade Federal do Rio Grande do Sul (UFRGS), Av. Sarmento Leite 500/305, Porto Alegre, RS 90050-170 Brazil; 5 Programa de Pós-Graduação em Bioexperimentação, Faculdade de Agronomia e Medicina Veterinária, Universidade de Passo Fundo (UPF), BR 285, São José, Passo Fundo, RS 99052-900 Brazil

## Abstract

The ability to avoid and escape from predators are clearly relevant behaviors from the ecological perspective and directly interfere with the survival of organisms. Detected in the aquatic environment, risperidone can alter the behavior of exposed species. Considering the risk of exposure in the early stages of life, we exposed zebrafish embryos to risperidone during the first 5 days of life. Risperidone caused hyperactivity in exposed larvae, which in an environmental context, the animals may be more vulnerable to predation due to greater visibility or less perception of risk areas.

## Introduction

Emotional states such as fear and anxiety are possible to be observed in zebrafish larvae. With a comprehensive behavioral range, these animals are already responsive to the environment at 24 hpf (hours post-fertilization) and within a week of life, they already respond to stimuli such as touch, sound, water movement or changes in light^1,2^. Unlike adults, zebrafish larvae prefer clear areas (dark areas are thought to simulate predator shade), avoid areas of light oscillation and recognize when placed in open areas^2–4^. Even during the first week of life, zebrafish larvae may already are sensitive to the same anxiolytics used for anxiety in humans^3,5^.

Behavioral changes have the potential to impact directly on the physical condition and the perpetuation of a species^2,4^. One of the most known behaviors in nature is the prey-predator relationship. To avoid predators and consequently potential life risks, the escape behavior is fundamental to the species maintenance. Emotions such as fear or anxiety can be observed in all vertebrates and are very important to maintainance and survival of the species, and the preservation of these escape patterns is observed in most fish species. Changes or alterations in this response may induce a direct risk to the individuals or even in severe populational consequences^1,3^.

In this context, aquatic contaminants are involved in many behavioral changes of exposed species. Several  drugs consumed by the population promotes an increase in the amount of drug residues in the aquatic environment^6–9^. Even when detected in low amounts, the ability to cause changes in the physiology of non-target organisms has not yet been fully elucidated, but it is known that even at low concentrations (ng/L or μg/L) these drugs may have effects on exposed species^6,8–10^.

Risperidone (RISP), an atypical antipsychotic used mainly for the treatment of schizophrenia and bipolar mood disorder, has already been detected at different levels in aquatic environments^11,12^. RISP levels have already been recorded up to 0.0014 μg/L in seawater^8^, 0.0029 μg/L in effluent water^7^, and 0.0034 μg/L in drinking water^13^. In Belgium, the highest level of environmental contamination was recorded, presenting 0.364 μg/L in affluents and 0.154 μg/L in Dendre River effluent^14^. A few studies have been carried out to find out the consequences of species exposure and this type of contaminant at low concentrations^15,16^. Despite behavioral changes are common findings in mammals exposed to risperidone during embryonic development^17^, no reports about behavioral changes in the RISP-exposed fish were found in the current literature. Thus, in view of the great importance of preserving all the behavioral repertoire, we sought to identify the effects of RISP exposition on behavioral parameters in embryos and larvae zebrafish.

## Results

**Figure 1:**
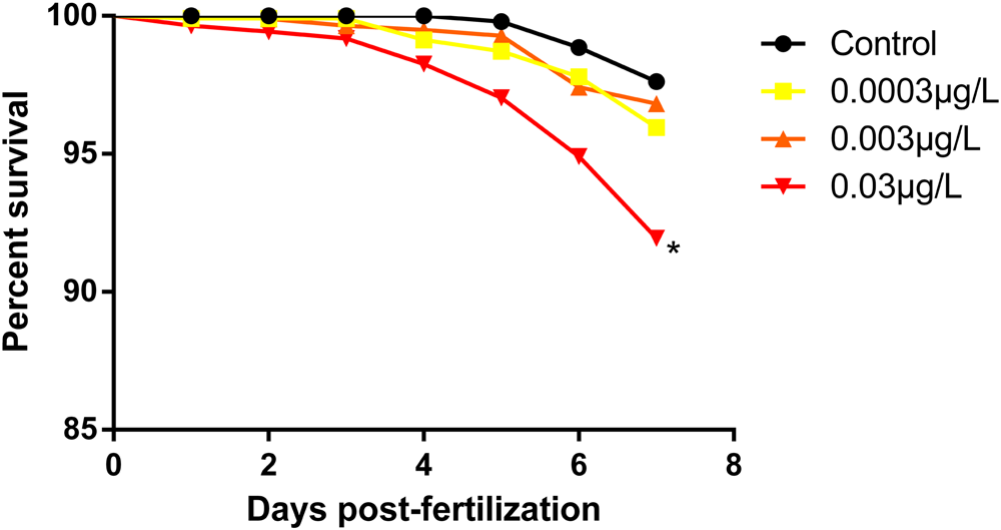
The larvae exposed to the highest concentration of risperidone tested showed increased mortality. Survival curve evaluated by Kaplan-Meier method. *p < 0.05. N = 160.

Fish exposed to the concentration of 0.03 μg/L showed an increase in mortality when compared to the control group (Fig. 1) (p < 0.0001). No differences were found in  hatching and heart rate.

**Figure 2:**
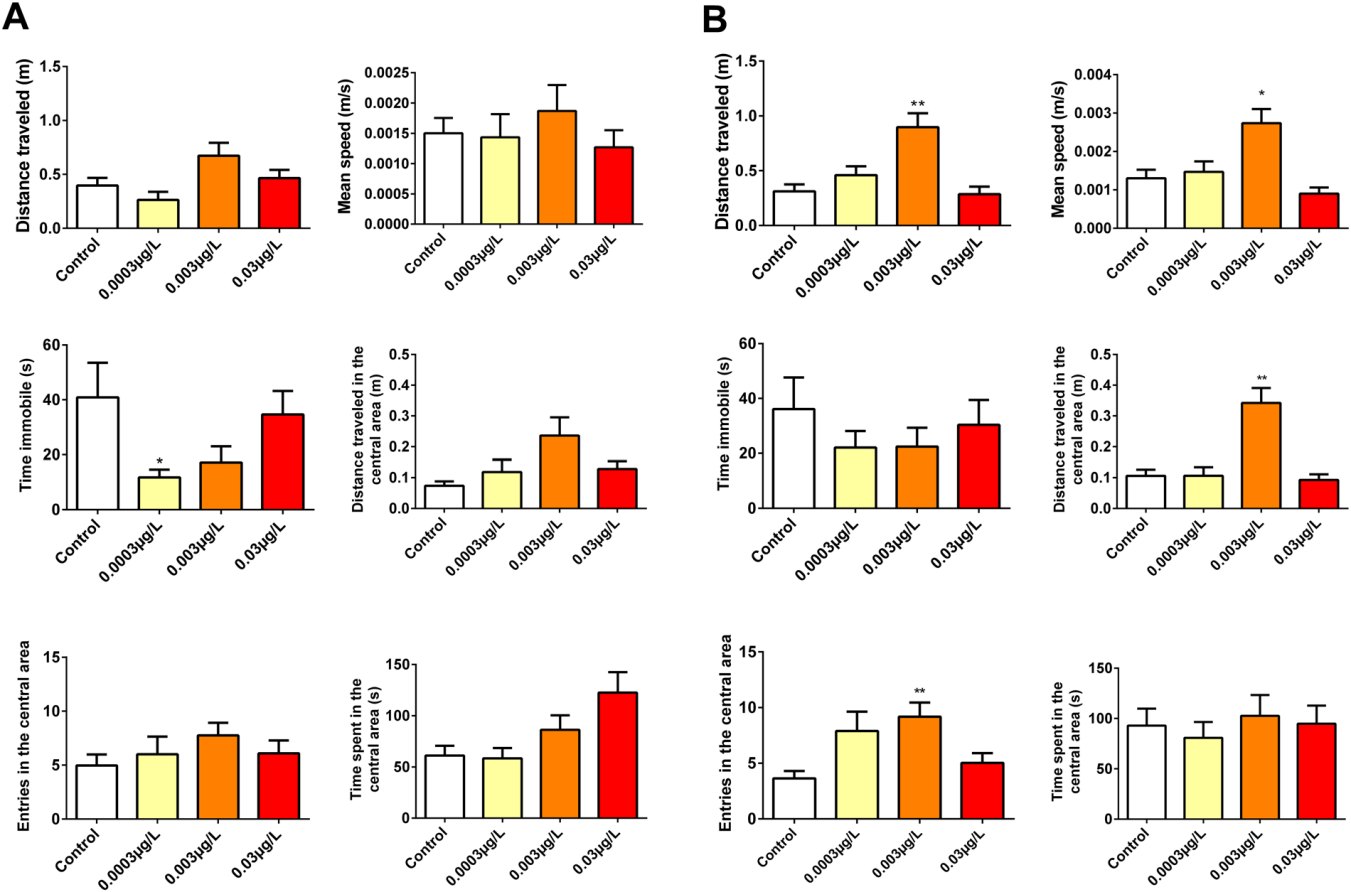
Open field test results. (**A**) The first phase (0–6 min). The concentration of 0.0003 µg/L RISP increased the immobility time of the exposed larvae. (**B**) The second phase (6–12 min). Hyperactivity can be observed by an increase in the distance, average speed and a number of entries in the central area. Means were compared by One-way ANOVA followed by Dunnett’s or Kruskal-Wallis test followed by Dunn’s were used depending on data normality. *p < 0.05. N = 30.

During the open field test, RISP at a concentration of 0.003 μg/L increased the total distance traveled (p = 0.0009), the number of entries in the central area (p = 0.0127) and the mean speed  in the final phase of the test (6–12 min) (p = 0.0009). The concentration of 0.0003 μg/L increased the immobility time in the settling phase (p = 0.0542) (0–6 min), but this result did not last during the final test phase (6–12 min) (p = 0.6093) (Fig. 2).

**Figure 3:**
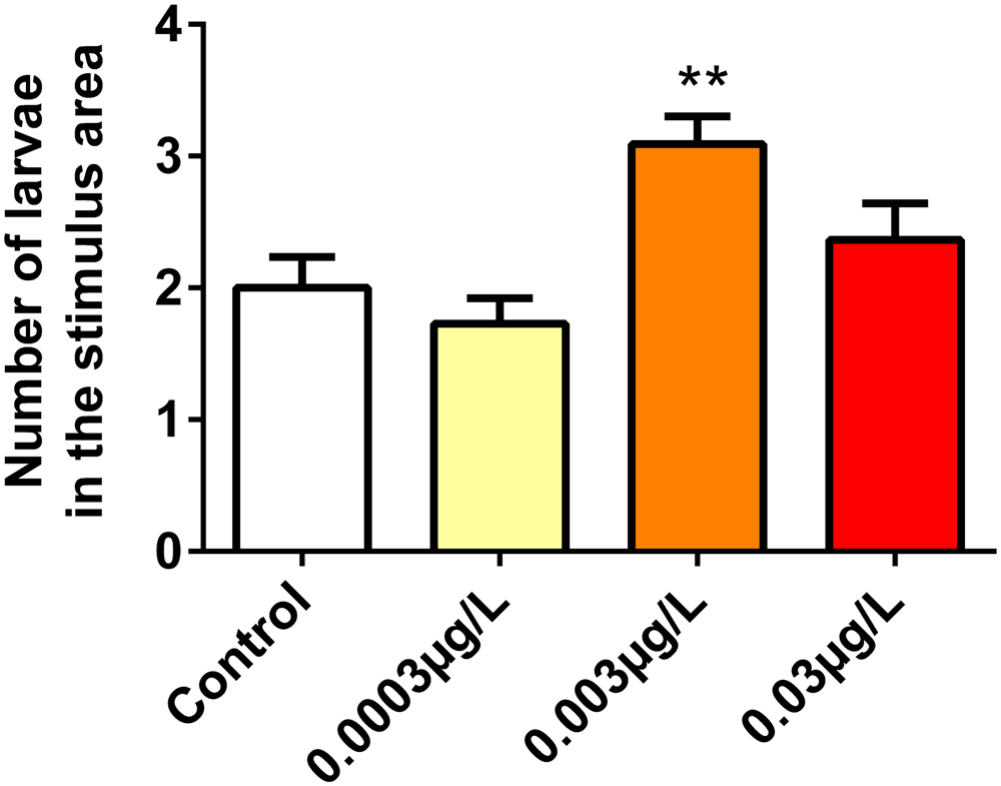
The concentration of 0.003 µg/L risperidone increased the number of animals that remained in the stimulus area. Means were compared by One-way ANOVA followed by Dunnett’s. *p < 0.05. N = 55.

Larvae exposed to the concentration of 0.003 μg/L RISP decreased the response to the aversive stimulus and remained mostly in the stimulus area when compared to the control group (p = 0.0011). The other groups did not present alterations (Fig. 3).

**Figure 4:**
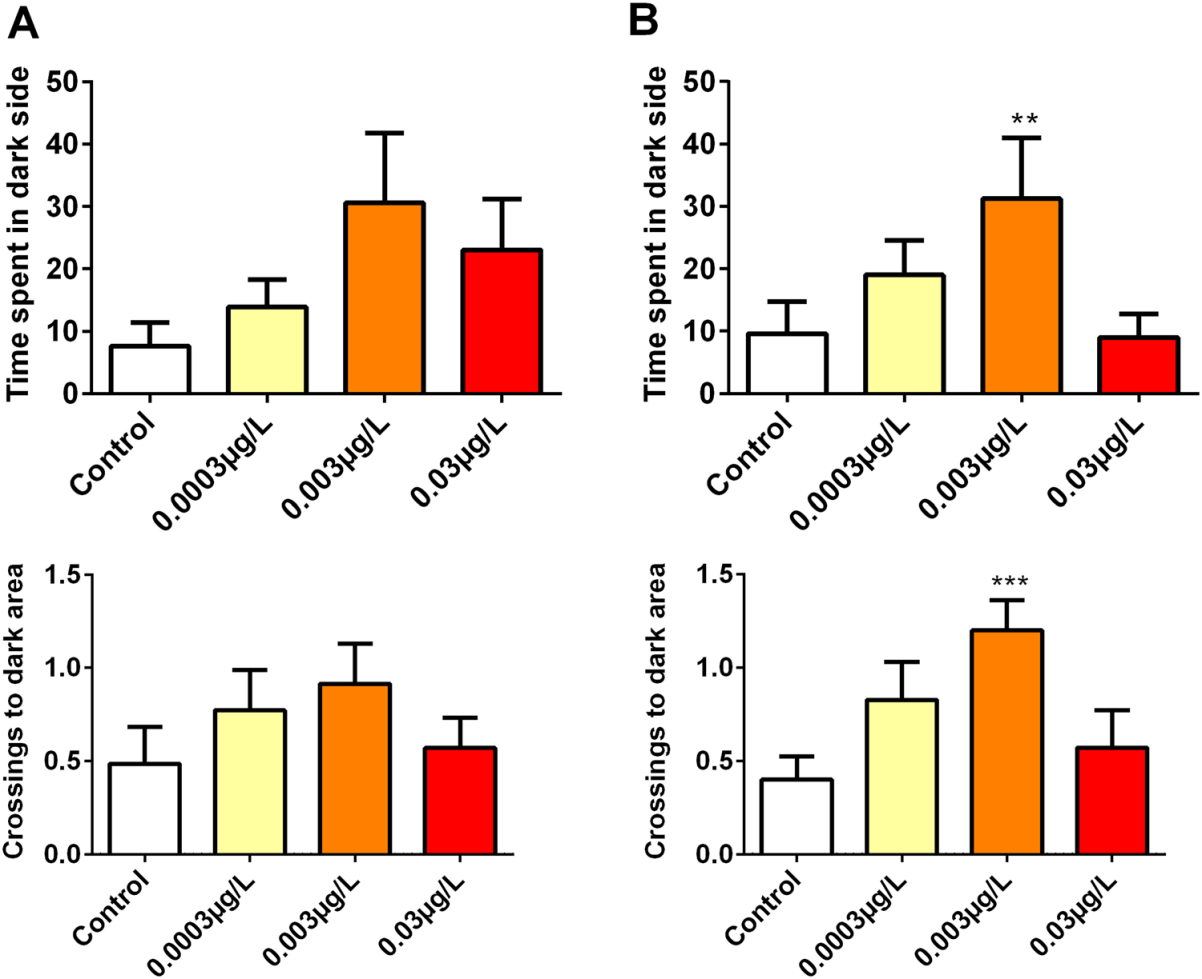
During the LDT, larvae exposed to the intermediate concentration of risperidone increased the time on the dark side and the number of crosses of the center line. Means were compared by One-way ANOVA followed by Dunnett’s or Kruskal-Wallis test followed by Dunn’s were used depending on data normality. *p < 0.05. N = 35.

At the concentration of 0.003 μg/L larvae decreased the latency for entry into the dark side of the well (p = 0.0229), as well as increasing the number of entrances and residence time on this side during the second phase of the test (6–12 min) (p = 0.1532). In the initial phase of the test, there was no difference between groups (Fig. 4). There was no difference between the groups tested in relation to spontaneous movement.

## Discussion

Here we show that the presence of RISP residues in water can alter the exploratory behavior of zebrafish embryos and larvae. In fact, during the first-time window (0–6 min) in the open field test, aversive stimuli and light/dark tests, larvae exposed to 0.0003 µg/L RISP increased the immobility time. The observed effects of extremely low RISP concentration were surprising since that this concentration was already detected in natural aquatic environments^13^, indicating the potential risk for populations exposed to this type of contaminant^10^. Moreover, larvae exposed to higher concentration of RISP displayed an increased mortality (5.68% in relation to the control group) and no significant alteration in hatching. These results are consistent with our previous study, where RISP affects parameters such as survival, hatching, heart rate and total larval length^16^.

In the 2^nd^ time window (6–12 min) of the exploration tests, larvae presented increased exploratory activity and decreased response to the environmental stimuli, both indicating hyperactivity. The difference between 1^st^ and 2^nd^-time window contradicts the actual knowledge that larvae do not present an adaptation period as seen in adults^18^. Further studies should be carried out to validate this hypothesis.

RISP is an atypical antipsychotic antagonist of serotonin and dopamine receptors^19^. Changes in the exploratory activity caused by RISP are common findings in adult rats^17^ and adult^20^ as well as larvae^21^ zebrafish. These changes in exploratory behavior are expected since that activity of dopaminergic and serotonergic neurons is related to motor coordination^22,23^.

The effects observed in our larvae appear to be associated with RISP exposure in early stages of development. In fact, the exposure to antipsychotics during the embryonic stage is associated with reduced levels of neurotransmitters leading to functional problems in exposed fish^24^ and mammals^17,25,26^. In addition, our larvae were exposed for 5 days and evaluated on the 6^th^ day. This 24h-period without exposure may be related to these effects since the chronic administration of psychoactive drugs also appears to lead to an increase in the activity of dopaminergic and serotonergic receptors shortly after the drug withdrawal^25–31^.

In our study, the effects were mainly observed in the intermediary RISP concentration, but not in the higher. In fact, our tested concentrations were lower than plasma levels suitable to cause therapeutic effects^32^ or concentrations commonly tested in scientific experiments^17,24,30,31^ but exerted effects on zebrafish larvae, like those described with higher concentration. Thus, any comparison between our results and those reported in the literature is difficult, since no reports were found using RISP concentration as low as we used in the present study.

The last comment is about the possible implications of our results. In fish, dopamine and serotonin are involved in locomotion, attack/defense, learning/memory and eating behavior^33^. The ability to capture prey and escape from predators are clearly relevant behaviors from the ecological perspective, as they directly interfere with the growth and survival of organisms^33,34^. A prey may be more susceptible to predation as a result of non-detection of predators, poor escape performance, reduced resistance, inability to learn and greater visibility due to hyperactivity^33,35^. Our results highlight that RISP altered larvae activity patterns, which in an environmental context can directly influence the ability to avoid or evade predatory behavior which may result in significant repercussions on the maintenance of the species as well on the ecosystem.

## Materials and Methods

### Ethical Aspects

This study was approved by the Animal Use Ethics Committee (CEUA) of the University of Passo Fundo, UPF, Passo Fundo, RS, Brazil (Protocol #9/2015) and complied with the guidelines of the National Council for Animal Experimentation Control (CONCEA). Approximately 600 larvae were tested during this experiment.

### Study strategy

We exposed embryos and larvae of zebrafish (*Danio rerio*, wild-type) to different concentrations of RISP already detected in the aquatic environment analyzing eventual changes in the larvae behavioral repertoire. This analysis was based on the different behavioral tests as follows: spontaneous movement, open field, light/dark as well as aversive stimulus. We opted for a 5-day chronic exposure to RISP since this period window correspond to the whole period of zebrafish organogenesis (2hpf to 120hpf)^36^.

### Reproduction and maintenance of embryo

For breeding, healthy zebrafish wild-type, aged between 3 and 18 months  were used. The animals were placed in barred bottom aquaria in ratios of 1:1 (males: females). After 12 hours of dark, during the morning the lights were on and after 1 hour the embryos were collected^36,37^. The methods of reproduction and maintenance of embryos are described in the previous work^16^.

After collection, the embryos were washed and classified as fertilized and unfertilized with the aid of light microscopy^37,38^. Embryos were maintained in E3 medium (reverse osmosis water + 60 mg/L Marine Ocean Instant Ocean) and distributed in 24 well cell culture plates (3 ml/well), 10 embryos per well and incubated in a water bath at 28 °C^39^. For the tests, embryos of up to 3hpf were accepted. The embryos were exposed to RISP from 3hpf to 120hpf.

### Concentrations tested

The RISP concentrations were based on those already registered in the aquatic environment: 0.00034 µg/L^13^, 0.003 µg/L, and 0.03 µg/L. These concentrations were previously tested and changed survival, hatching, heart rate and total larval length^16^. The solutions were prepared and stored in amber glass bottles, where they remained heated in a water bath to be replenished in the wells when necessary.

### Survival and hatching analysis

For analysis of survival and hatching, we have monitored all animals once a day in the morning for 7 days with the aid of a magnifying glass or optical microscopy. Embryos and larvae that do not show transparency, coagulated or without cell formation, cardiac movement or blood circulation were considered dead. Animals were considered “hatched” when partially or completely outside of the chorion. For this hatching and survival measurements, we analyzed 160 embryos by concentration (control and the three concentrations tested), totalizing 640 embryos

### Spontaneous movement

In 24 hpf the embryos present spontaneous movements of the tail still inside the chorion. They are thus considered because they are induced by the development of the motoneurons without any control by the central nervous system^37,40,41^. These movements were recorded in 1 minute^40^ in 64 embryos by group (total of 256 embryos).

### Heart rate

Heart rate was assessed at 48hpf in all groups during the morning. The heart rate was manually counted by light microscopy for 1 minute^42^ in 48 embryos by group (total of 192 embryos).

### Open field test

To perform the open field test, the larvae at 6 dpf were placed in 10 mL wells containing only E3 medium and filmed (Canon EOS Rebel T5 Macro Lens EF 100mm) for 12 minutes. Similar to the behavior seen in mammals, zebrafish larvae also present thigmotaxis and recognize when placed in a new environment^3,4,43^.

**Figure 5:**
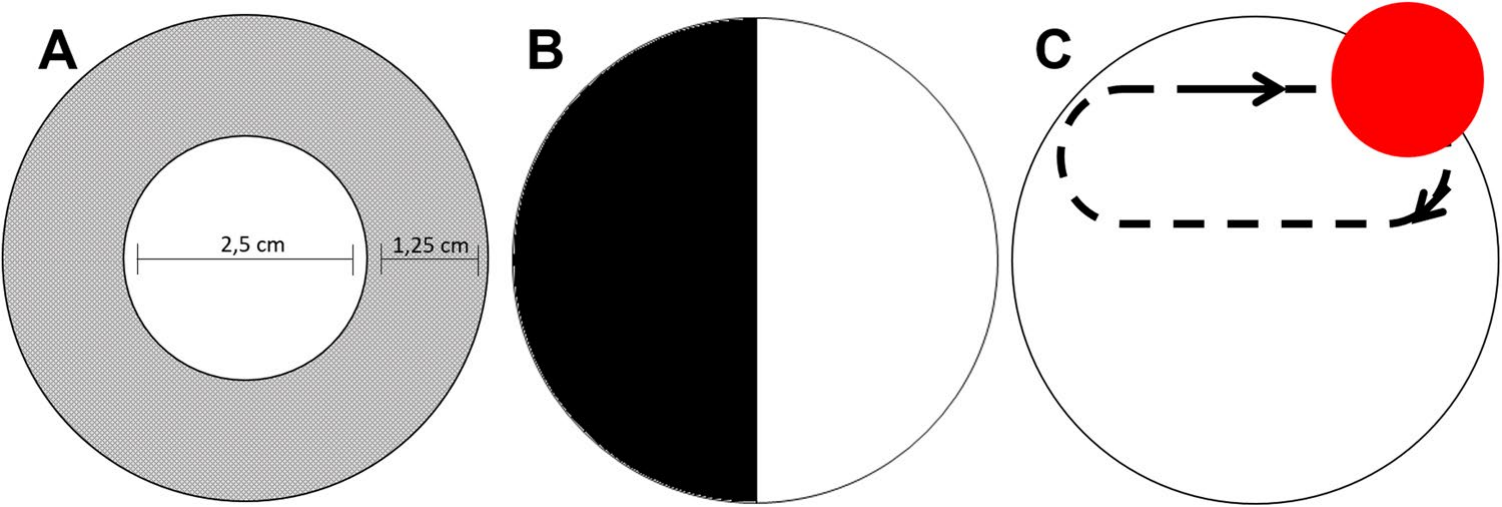
A schematic illustration of the experimental setup used to record zebrafish larvae behavior. (**A**) Open field test - Central and peripheral region established in the software ANY-Maze (**B**) Light/dark test (**C**) Aversive stimulus was produced with a red bouncing-ball.

For thigmotaxic behavior analysis, we filmed 30 larvae by group (total of 120 larvae). In the videos, the well was virtually divided into a central and peripheral area (Fig. 5A)^18^ and the period were divided into two phases: adaptation period (0–6 min) and the exploratory period (6–12 min). ANY-maze software was used to analyze the following parameters: total distance travelled, time in the central area, distance travelled in the central area, entries in the central area, immobility time and mean speed.

### Light/dark test (LDT)

For this test, a 6-well cell culture plate was used with one well (5 ml) divided into a dark (black) area and a clear (white) area. Unlike adults, zebrafish larvae prefer to stay in the white area of the well (Fig. 5B). It is believed that the dark area represents the shadow of a possible predator^43,44^.

Thirty-five larvae by group (total of 140 larvae) were placed in the test area and filmed for 6 minutes. The latency for entry into the dark side, the number of entries and dwell time on the dark side were evaluated using the ANY-maze software. As in the open field test, the LDT was divided between the initial phase (0–6 min) and final phase (6–12 min).

### Aversive stimulus test (AST)

Aversive stimulus aims to test the cognitive ability of the larva to identify areas of danger. Tests with colorations have been used for their ecological relevance, since different species of fish, like zebrafish, use colors to differentiate possible foods, recognize specifics as well as avoid predators^18^.

For this test, the larvae were placed in 6-well cell culture plates (5 larvae per well, n = 55 by group totalizing 220 larvae) above an LCD monitor. After the adaptation period (2 min), using PowerPoint software (Microsoft Office Professional Plus 2013), we started the exposure to a visual stimulus area with a red sphere of 1.35 cm in diameter with a trajectory that traveled only half the well (Fig. 5C). The animals were stimulated for 5 min and at the end of the test were recorded the number of animals that remained in the stimulus area and those that remained in the non-stimulated area^45^.

### Statistical analysis

For statistical analysis and graphing we used GraphPad Prism software version 6.01 for Windows. Survival and hatchability data were evaluated by Kaplan-Meier method. For the analysis of the heart rate data, spontaneous movement, open field test, LDT and AST, One-way ANOVA followed by Dunnet’s (a group of parametric data ) or Kruskal-Wallis test followed by Dunn’s were used (a group of non-parametric data). All groups were compared to the control group. Statistical significance was accepted when p ≤ 0.05.
